# Refolding of bioactive human epidermal growth factor from *E. coli* BL21(DE3) inclusion bodies & evaluations on its *in vitro* & *in vivo* bioactivity

**DOI:** 10.1016/j.heliyon.2022.e09306

**Published:** 2022-04-18

**Authors:** Iman Permana Maksum, Yosua Yosua, Ahmad Nabiel, Riyona Desvy Pratiwi, Sriwidodo Sriwidodo, Ukun M.S. Soedjanaatmadja

**Affiliations:** a Department of Chemistry, Faculty of Mathematics and Natural Science, Universitas Padjadjaran, Sumedang, Indonesia; b Research Centre of Biotechnology, Indonesian Institute of Science, Bogor, Indonesia; c Department of Pharmaceutics & Pharmaceutical Technology, Faculty of Pharmacy, Universitas Padjadjaran, Sumedang, Indonesia

**Keywords:** *E. coli* BL21(DE3), Heat-resistant, hEGF, Freeze-thawing, Glutathione-based redox system

## Abstract

Human epidermal growth factor (hEGF) is a mitogenic protein widely used in pharmaceutical and cosmetic industries, thus recombinant DNA technology has been applied to meet the high demand for hEGF. The overexpression of recombinant protein in *E. coli* often leads to the formation of inclusion bodies (IBs). Mild solubilisation preserves the native secondary protein structure in IBs, thereby the high recovery of active protein from IBs. The redox system also plays a pivotal role in the formation of disulphide bonds during refolding of disulphide bond-containing protein. This study aimed to recover hEGF from bacterial IBs through freeze-thawing solubilisation and glutathione-based oxidative refolding. CBD-*Ssp* DnaB-hEGF fusion protein was expressed as IBs in *E. coli*, washed with Triton X-100 and urea to remove most protein contaminants, then the solubilised fusion protein was obtained by freeze-thawing with the addition of 2 M urea. The solubilised protein was subsequently refolded by intein cleavage via a glutathione-based redox system. The refolded hEGF demonstrated heat-resistant properties, interacted with specific antibodies on ELISA, stimulated keratinocyte proliferation and possessed significant *in vivo* wound healing properties on the 8^th^ day, confirming that hEGF was correctly folded. In summary, the protocol described is suitable for the recovery of refolded hEGF from bacterial IBs by mild solubilisation and oxidative refolding.

## 1 Introduction

Human epidermal growth factor (hEGF) is a 6.2 kDa protein consisting of 53 amino acid residues and three intramolecular disulphide bonds (Tang et al., 2016; Zheng et al., 2016). It promotes cell proliferation, differentiation, and migration (Carpenter and Cohen, 1990; Schultz et al., 1991), exhibits mitogenic properties in accelerating the wound healing process and is effective in preventing skin ageing (Sriwidodo et al., 2020). It is also used as a therapeutic protein in the pharmaceutical and cosmetic industries (Aldag et al., 2016; Sriwidodo et al., 2019). To meet the high demand for hEGF, some studies have attempted to increase hEGF production, with recombinant DNA technology being the most commonly used approach (Indriyani et al., 2019; Sriwidodo et al., 2017), as it provides a large amount of recombinant protein and low production cost (Merlin et al., 2014).

In recent years, hEGF has been produced in several expression systems such as *Escherichia coli* (Maksum et al., 2017), *Pichia pastoris* (Eissazadeh et al., 2017), *Saccharomyces cerevisiae* (George-Nascimento et al., 1988), transgenic plants (He et al., 2016), and mammalian cells (Negahdari et al., 2016). The *E. coli* expression system has attracted the attention of many researchers as it is relatively cheap, easy, and fast for recombinant protein production (Hayat et al., 2018). It also offers a high cell density in a short time as well as a high expression rate of recombinant protein (Bahreini et al., 2014; Silaban et al., 2019). However, the high expression rate often leads to the formation of inclusion bodies (IBs) (Gomes et al., 2016), misfolded and partially folded protein which form insoluble aggregates through hydrophobic interactions (de Groot et al., 2008). The IBs are produced in large amounts, homogeneous, easy to isolate, resistant to proteolysis, and have a native-like secondary structure (Singh et al., 2015; Singh and Panda, 2005).

Many studies have shown that bioactive protein can be obtained from IBs by restoring its structure into a native state involving several steps including isolation of IBs, solubilisation, refolding of the solubilised protein, and purification of the refolded protein (Singhvi et al., 2020). Solubilisation of IBs and refolding of the solubilised protein into a native state are the most important steps in recovering bioactive protein from IBs (Singh et al., 2015). Mostly, IBs are solubilised using a high concentration of urea or guanidine hydrochloride (Clark, 2001) to unfold the protein structure and expose the hydrophobic patch causing the formation of aggregates, thus resulting in a low recovery of bioactive protein (Upadhyay et al., 2014; Yon et al., 2018). Mostly, IBs are solubilized using high concentration of urea or guanidine hydrochloride (Clark, 2001). They will unfold the protein structure and expose the hydrophobic patch. The usage of high urea concentration is often resulting in low recovery of bioactive protein due to the formation of aggregates during refolding step (Upadhyay et al., 2014; Yon et al., 2018). Solubilisation of IBs using milder conditions can retain the native-like secondary structure, thus making it easier to restore the structure of solubilised protein into the native state, reducing protein aggregation during refolding and increasing bioactive protein recovery (Sahdev et al., 2008; Upadhyay et al., 2016). Therefore, mild solubilisation is a key strategy for increasing bioactive protein recovery.

Different mild solubilisation methods have been developed such as the use of low concentrations of urea (Patra et al., 2000), alkaline pH (Singh et al., 2008), organic solvent (Singh et al., 2012), and high hydrostatic pressures (Chura-Chambi et al., 2008). Qi et al. (2015) described a single freeze-thawing process for mild solubilisation of IBs, in which, the formation of ice crystals in the presence of a low concentration of urea promoted IBs solubilisation. This method is simple and flexible for the solubilisation of various proteins (Qi et al., 2015). The solubilised protein will start to refold while the denaturant concentrations decrease (Yamaguchi and Miyazaki, 2014) and the denaturant can be removed by dilution, dialysis or a solid-phase method (Yamaguchi et al., 2013). The formation of proper disulphide bonds is important during the refolding of disulphide bond-containing proteins like hEGF (Han et al., 2012; Wang et al., 2006). An oxido-shuffling agent is added to the refolding buffer to promote disulphide bond exchange and the formation of disulphide bonds, thereby increasing the formation of native disulphide bonds (Moghadam et al., 2015; Thomson et al., 2012). Widely used oxido-shuffling agents are a combination of glutathione in reduced (GSH) and oxidised (GSSG) form (Thomson et al., 2012), which has been used to refold disulphide bond-containing proteins such as antibodies (Sun et al., 2014), enzymes (Mirhosseini et al., 2019), and growth factors (Gieseler et al., 2018). Hence, the addition of GSH and GSSG during refolding of hEGF will assist in the formation of the native disulphide bond.

This study aimed to recover hEGF from bacterial IBs through freeze-thawing solubilisation and glutathione-based oxidative refolding. The hEGF was expressed as CBD-*Ssp* DnaB-hEGF fusion protein in the form of IBs using an *E. coli* expression system, solubilised by freeze-thawing, then refolded with the addition of the glutathione-based redox system. The *Ssp* DnaB mini-intein cleavage was induced by dialysis with a pH gradient. The refolded hEGF demonstrated heat resistance properties, exhibited specific binding on ELISA, stimulated *in vitro* cell proliferation and *in vivo* wound healing, thus the protocol described is suitable for the recovery of refolded hEGF from bacterial IBs.

## 2 Materials & methods

### 2.1 Bacterial strain, plasmid and reagents

*E. coli* BL21(DE3) harbouring the recombinant plasmid pD861-CBD-*Ssp* DnaB-hEGF [3070 bp]was used as a host for CBD-*Ssp* DnaB-hEGF expression. The recombinant plasmid was constructed by our team and synthesized by ATUM (Newmark, CA, USA). The CBD-*Ssp* DnaB-hEGF was successfully expressed by Yosua et al. (2021) as a 31.4 kDa protein. Ammonium persulphate, coomassie brilliant blue G-250, 3-(4,5-dimethylthiazol-2-yl)-2,5-diphenyl-2H-tetrazolium bromide (MTT) reagent, sodium chloride, urea, Tris base, and β-mercaptoethanol were purchased from Merck (USA). Acrylamide/bis solution, molecular weight marker, and Tetramethylethylenediamine (TEMED) were purchased from Bio-Rad (USA). EDTA disodium salt was purchased from 1^st^ base (Singapore). Yeast extract was obtained from Oxoid (UK). Dialysis tubing (MWCO 3 kDa), kanamycin sulphate, L-rhamnose, recombinant hEGF, tryptone, tricine, and Triton X-100 were purchased from Sigma-Aldrich (Singapore). Oxidised (GSSG) and reduced glutathione (GSH) were purchased from Tokyo Chemical Industry (Japan). Anti-EGH monoclonal antibody and alkaline phosphatase-conjugated anti-mouse IgG antibody were purchased from Santa Cruz Biotechnology (USA). LB medium was prepared from 1% (w/v) sodium chloride, 1% (v/v) tryptone, and 0.5% (v/v) yeast extract.

### 2.2 Expression of CBD-*Ssp* DnaB-hEGF as IBs in *E. coli* BL21(DE3)

A frozen glycerol stock of *E. coli* BL21(DE3) [pD861-CBD-*Ssp* DnaB-hEGF] was inoculated in 5 mL Luria Bertani (LB) medium supplemented with 50 μg/mL kanamycin, then incubated at 37 °C overnight with shaking at 200 rpm. The overnight culture (1 mL) was transferred to 100 mL LB medium supplemented with 50 μg/mL kanamycin, then incubated at 37 °C with shaking at 200 rpm until the OD_600_ reached 0.6. L-rhamnose was added to a final concentration of 4 mM to induce protein expression and incubated for 5 h. The culture was harvested, centrifuged at 6,000 *g* for 20 min at 4 °C and the cell pellet was stored at -20 °C for further analysis. The Tricine SDS-PAGE procedure was performed according to Haider et al. (2012) with total amount of sample loaded were 20 μL into each well.

### 2.3 Isolation of CBD-*Ssp* DnaB-hEGF inclusion bodies from the cell pellet

The cell pellet was resuspended in 1:4 (w/v) lysis buffer (20 mM Tris-Cl, 1 mM EDTA, pH 8.5) and sonicated in an ice bath for 1 min with 1 min gap for 10 cycles (1 s on, 1 s off pulse), then centrifuged at 12,000 *g* for 20 min at 4 °C. The IBs were washed twice with 1:8 (w/v) washing buffer I (20 mM Tris-Cl, 1 mM EDTA, 1 M urea, 1% Triton X-100, pH 8.5), centrifuged for 12,000 *g* for 12 min at 4 °C. The washing step was repeated, once with washing buffer II (20 mM Tris-Cl, 1 mM EDTA, 1% TritonX-100, pH 8.5) and twice with washing buffer III (20 mM Tris-Cl, pH 8.5). The purified IBs were then subjected to solubilisation.

### 2.4 Solubilisation of purified inclusion bodies by freeze-thawing

#### 2.4.1 Solubilization efficiency in different concentrations of urea

The purified IBs were solubilised using the method previously described by Qi et al. (2015) with some modifications. Briefly, the IBs were resuspended in 1:8 (w/v) solubilization buffer (20 mM Tris-Cl, pH 8.5) containing different concentrations of urea (0–8 M), incubated at -20 °C overnight, then thawed at room temperature the next day. The mixture was centrifuged at 12,000 *g* for 20 min at 4 °C and the supernatant was separated from the insoluble aggregate. A urea denaturing method was used as a comparison, whereby the suspension was incubated at room temperature for 2 h with shaking at 150 rpm, then centrifuged. The solubilised protein was characterised by 15% Tricine SDS-PAGE and the protein band was quantified by densitometry analysis using ImageJ software (María et al., 2020; Nguyen et al., 2017).

#### 2.4.2 Solubilization efficiency in different pH buffers

The overall solubilisation procedure was the same as described above using a solubilization buffer (20 mM Tris-Cl, 2 M urea) with different pH (8–12).

### 2.5 Refolding and self-cleavage of CBD-*Ssp* DnaB-hEGF

The solubilised CBD-*Ssp* DnaB-hEGF in solubilization buffer (20 mM Tris-Cl, 2 M urea, 5 mM β-mercaptoethanol, pH 12) was refolded by the drop-wise addition of 1:9 (v/v) refolding buffer (20 mM Tris-Cl, 100 mM NaCl, 100 mM sucrose, 2 mM GSH, 0.2 mM GSSG, pH 8.5) with constant stirring at room temperature for 4 h. The protein mixture was then transferred to dialysis tubing (MWCO 3 kDa) for intein cleavage by dialysis against 50 mM Tris-Cl, pH 7.5 and pH 6.0 for 24 h, respectively. The protein mixture was separated from the aggregate by centrifugation at 13,000 *g* for 10 min at 4 °C before the solubilised protein was characterised by 15% Tricine SDS-PAGE. The presence of hEGF in the sample was confirmed by western blot analysis following the protocol described previously by Latifah et al. (2020).

### 2.6 Characterisation of refolded hEGF

#### 2.6.1 Heat treatment

The mixtures of refolded protein were incubated at 60–85 °C for 20 min, then was centrifuged at 20,000 *g* for 10 min at 4 °C, the supernatant was decanted and characterised by Tricine SDS-PAGE.

#### 2.6.2 ELISA

The Qayee ELISA kit was used to evaluate the specific binding of the refolded hEGF. The analysis was performed and monitored at A_450_ according to the manufacturer's instruction.

#### 2.6.3 MTT assay

The refolded hEGF was tested to stimulate cell proliferation, in this experiment we compared hEGF activity with autologous serum (AS) and the combination of both in stimulating cell proliferation. Keratinocytes from human cell line HaCat were seeded in 96-well plates at density 2 × 10^4^ cell/mL and incubated for 24 h. The cell was cultured in RPMI-46 media containing 5% calf serum and 100 μg/mL kanamycin. The well plates were washed with 100 uL 1X PBS then subjected to 100 μL samples and incubated for 48 h in CO_2_ incubator. Next, the well plates were washed with 1X PBS before the addition of MTT reagent. The reaction was stopped by DMSO after 4 h then the absorbance was observed at 450 nm. The proliferation percentage was calculated using the following equation (Maksum et al., 2021):

(1) $$\text{\%Proliferation}=\frac{{\text{A}}_{\text{treatment}}}{{\text{A}}_{\text{without ​treatment}}}\times 100\text{\%}$$

### 2.7 Wound healing

#### 2.7.1 *In vivo* wound healing

A total of 20 male Swiss Webster mice weighing 20–30 g (around 6–8 weeks) were prepared for *in vivo* wound healing and acclimatised for 1 week before testing. The mice were kept in 25 °C with 82% humidity, with light/dark cycle for 12 h each. The mice were anaesthetised with an intraperitoneal injection of 50 mg/kg ketamine. The dorsal hair of mice was shaved and sterilised with 70% ethanol, then, a rounded wound was made using a 4 mm diameter biopsy punch. All experiments were performed following the guidelines of OIE animal welfare standards and were approved by the local Ethical Committee from Faculty of Medicine, Universitas Padjadjaran, Bandung (No: 1296/UN6.C10/PN2017). A thin film of hEGF-liposome-chitosan containing refolded hEGF (heat-treated fraction) was applied to the wound twice a day for 12 days. The thin film EGF-liposome-chitosan was prepared according to previous study (Umar et al., 2021). The experimental groups consisted of:

Group 1: Control without any treatment.

Group 2: Treated with a plain thin-film chitosan patch.

Group 3: Treated with a thin-film chitosan patch containing hEGF-liposome (75 μg/mL)

Group 4: Treated with a thin-film chitosan patch containing hEGF-liposome (100 μg/mL)

The wound was visualised using a “USB microscope” and the wound area was measured by ImageJ software on days 0, 4, 8, 10, and 12 after treatment. The percentage of wound closure was calculated using the following equation (Sriwidodo et al., 2020):

(2) $$\text{Wound ​closure ​}\left(\text{\%}\right)=\frac{\text{Initial ​wound ​area}-\text{Final ​wound ​area ​at ​a ​}\left(\text{t}\right)\text{ ​day}\left(\text{s}\right)\text{ ​after ​treatment}}{\text{Initial ​wound ​area}}\times 100\text{\%}$$

#### 2.7.2 Histopathology

The sample was taken from the middle of the wound from each group and fixed in 10% Neutral Buffered Formalin (NBF). The tissue was cut and placed on a tissue cassette, then dehydrated by automated dehydration apparatus and dried by vacuum. Clan was blocked by paraffin, then was cut into a 3–5 μm thick slice using a microtome. Later, the clan was placed on object glass and stained with Mallory-azan. The histopathologic cuts were observed under light microscope at 400x magnification.

### 2.8 Statistical analysis

For the wound healing activity test, the data were analysed and evaluated statistically using one-way analysis of variance (ANOVA). The software Statistical Package for Social Science (SPSS) was used to perform the statistical analysis and a *p*-value less than 0.05 was considered significant.

## 3 Results

### 3.1 Expression of CBD-*Ssp* DnaB-hEGF

**Figure 1:**
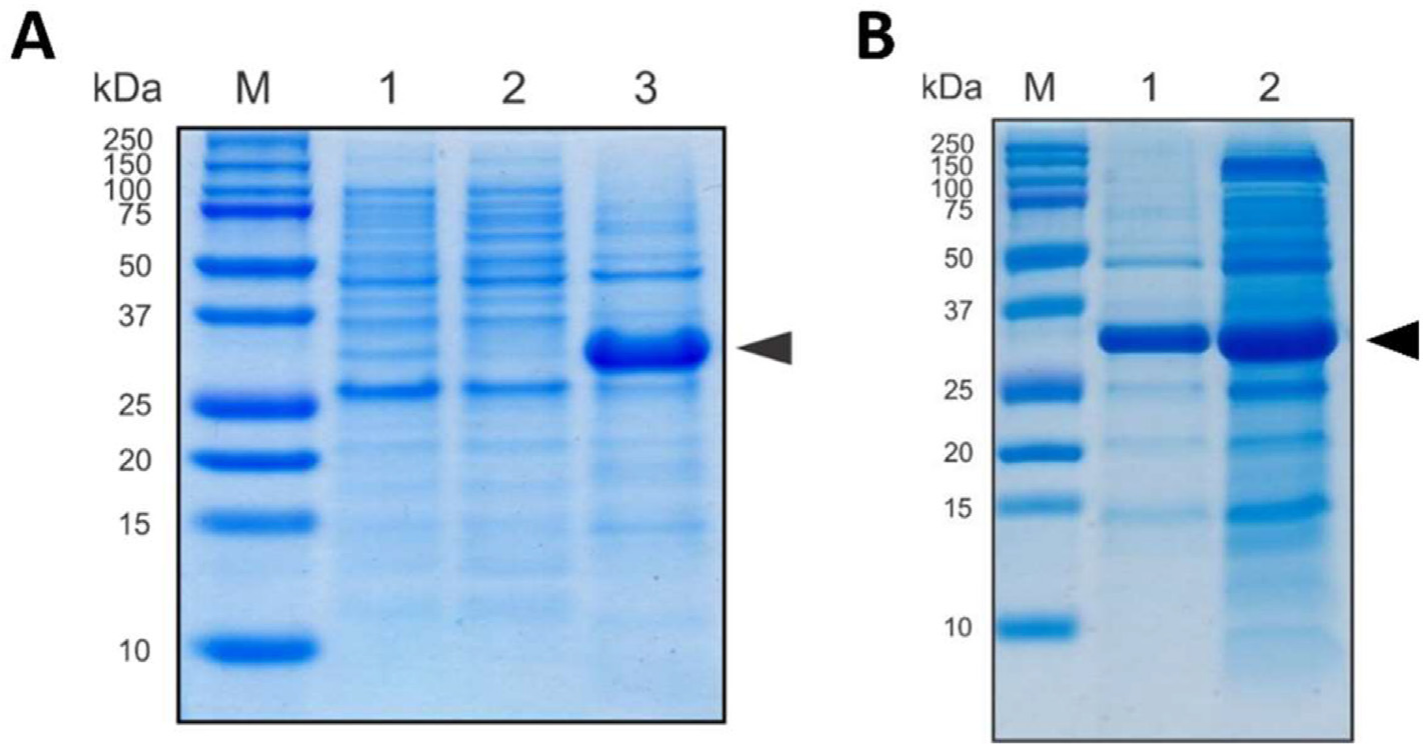
Tricine SDS-PAGE analysis of CBD-*Ssp* DnaB-hEGF expressionas IBs in *E. coli* BL21(DE3) (A). Lane M, protein marker; Lane 1, induced cell lysate; Lane 2, cytoplasmic fraction of induced cells; Lane 3, un-purified IBs of induced cells. Comparison of IBs before and after purification from expressed IBs for next solubilization step (B). Lane 1, purified IBs; Lane 2, un-purified IBs. Molecular weight of protein marker indicated on the left side. The black arrow indicates a 31.4 kDa CBD-*Ssp* DnaB-hEGF. The original, non-cropped gels were provided in supplementary data as Figure S1 & S2.

CBD-*Ssp* DnaB-hEGF was successfully expressed as a 31.4 kDa fusion protein using the pD861-CBD-Ssp DnaB-hEGF vector in *E. coli* BL21(DE3). As expected, it was found predominantly as IBs after cell lysis (Figure 1A), with little fusion protein detected in the soluble fraction due to *in vivo* splicing of *Ssp* DnaB mini-intein. The IBs were prepared by washing several times with buffer containing Triton X-100 and a low concentration of urea to remove most contaminants (Figure 1B). The insoluble pellet was mainly contaminated by cell debris and aggregated-cellular protein. Therefore, the contaminant did not interfere with refolding and fewer purification steps were necessary. The purified IBs were used for further solubilisation.

### 3.2 Solubilisation of IBs by single freeze-thawing

**Figure 2:**
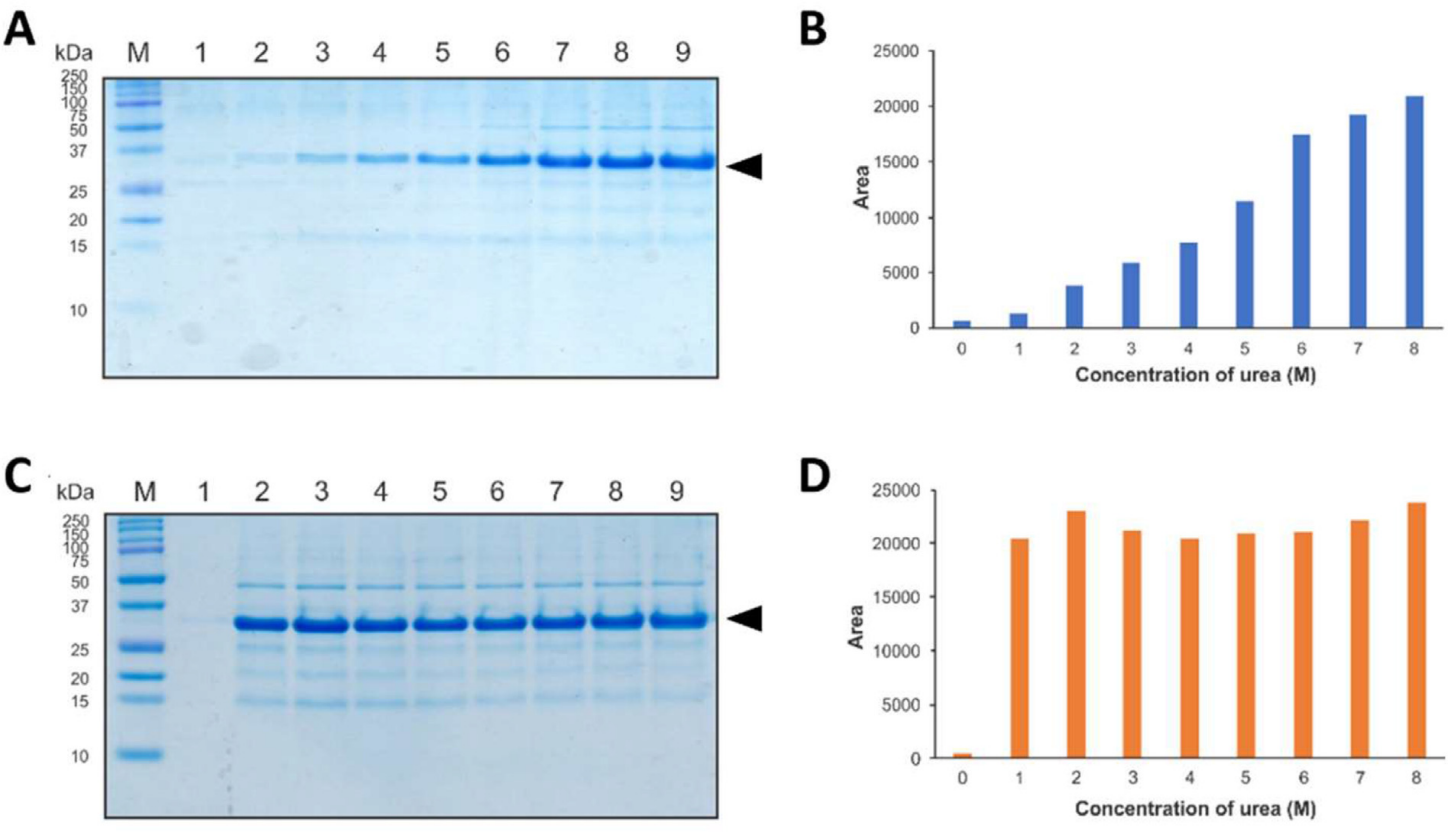
Solubilisation of IBs in Tris buffer containing different concentrations of urea. Tricine SDS-PAGE analysis of solubilised CBD-*Ssp* DnaB-hEGF by urea denaturing (A) and freeze-thawing (C) methods. Lane M, protein marker (MW indicated on the left side); Lane 1–9, solubilised CBD-*Ssp* DnaB-hEGF in different concentrations of urea (0–8 M). Target protein bands obtained by urea denaturing (B) and freeze-thawing (D) methods were quantified by ImageJ software. The black arrow indicates a 31.4 kDa CBD-*Ssp* DnaB-hEGF.

The potential of freeze-thawing for the solubilisation of CBD-*Ssp* DnaB-hEGF IBs was examined in the presence of different concentrations of urea and buffer pH using the urea denaturing method for comparison. The IBs could only be solubilised in the presence of 2–8 M urea (Figure 2 A & B), with more solubilised CBD-*Ssp* DnaB-hEGF obtained with increasing urea concentration. Regarding freeze-thawing, the Tris buffer containing 1–8 M urea effectively solubilised IBs (Figure 2 C & D), with most solubilised protein obtained in presence of 2 M urea. These result was in accordance with the study from Qi et al. (2015). Taken together, the results were indicated that the solubilised CBD-*Ssp* DnaB-hEGF obtained by freeze-thawing with 2 M urea was comparable to the urea denaturing method with 8 M urea.

**Figure 3:**
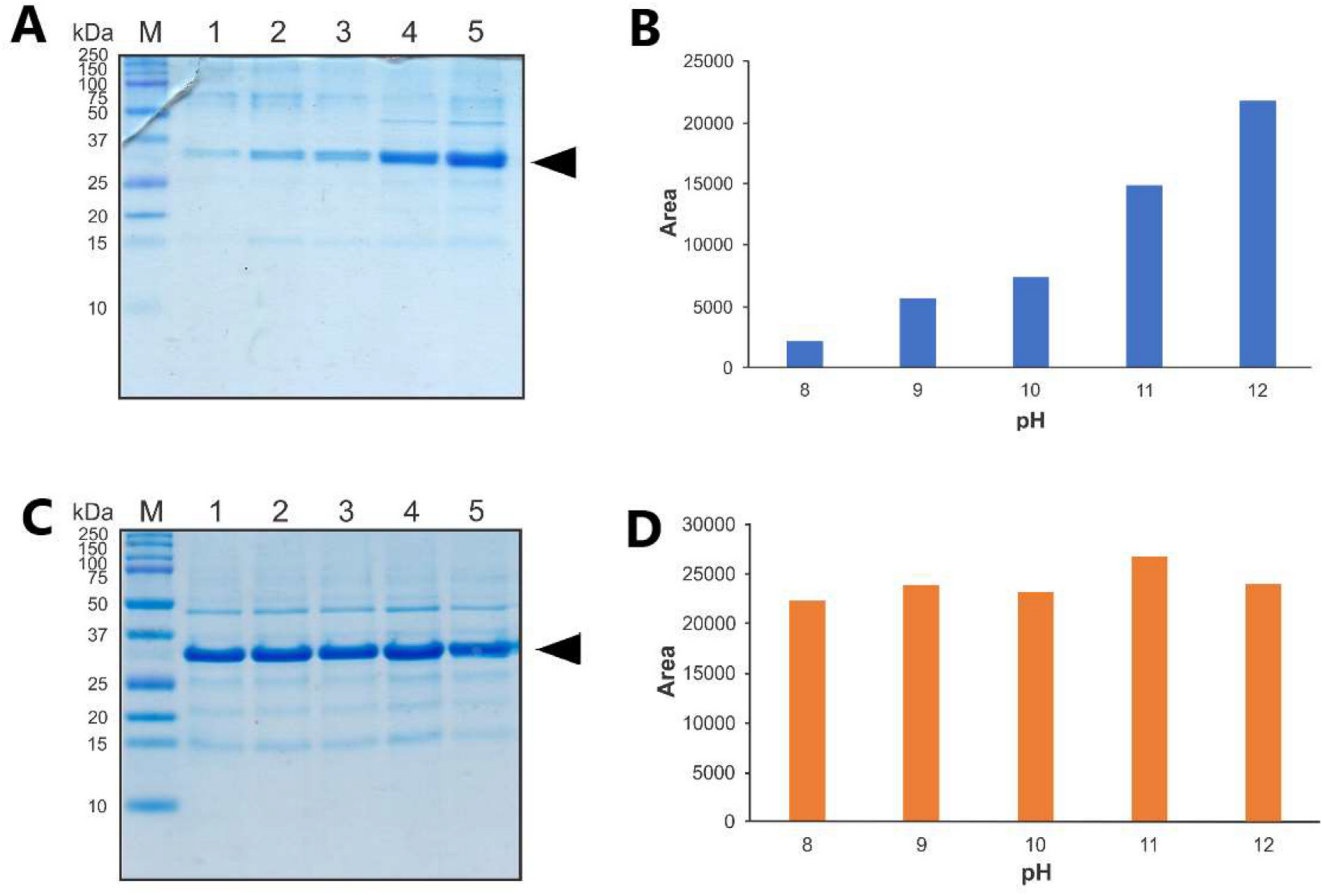
Solubilisation of IBs in different pH Tris buffer containing 2 M urea. Tricine SDS-PAGE analysis of solubilised CBD-*Ssp* DnaB-hEGF by urea denaturing (A) and freeze-thawing (C) methods. Lane M, protein marker (MW indicated on the left side); Lane 1–5, solubilised CBD-*Ssp* DnaB-hEGF in different pH Tris buffer (pH 8–12). Target protein bands obtained by urea denaturing (B) and freeze-thawing (D) methods were quantified by ImageJ software. The black arrow indicates a 31.4 kDa CBD-*Ssp* DnaB-hEGF.

The effect of pH on IBs solubilisation was also examined and to prevent uncontrolled *Ssp* DnaB mini-intein cleavage during solubilization, the minimum pH used was 8. It was known that *Ssp* DnaB mini-intein is expected to cleavage when the pH was lowered until reached pH 6–7, thus pH 8 was used as a starting point (Esipov et al., 2008; Zhang et al., 2015). The IBs were solubilised in Tris buffer containing 2 M urea with different pH (8–12). Under non-denaturing condition (2 M urea), the solubilized protein from IBs preserve its native-like structure, while under denaturing condition (8 M urea) it was fully denatured. It was well known that preservation of native-like structure of protein in IBs could leading to the higher refolded protein after refolding step (Singh et al., 2008, 2015). For the urea denaturing method, more solubilised CBD-*Ssp* DnaB-hEGF was obtained with increasing pH (Figure 3 A & B), with most solubilised CBD-*Ssp* DnaB-hEGF obtained in Tris buffer pH 12. In contrast, the same amount of solubilised CBD-*Ssp* DnaB-hEGF was obtained at each pH tested for the freeze-thawing method (Figure 3 C & D). Presumably, the pH used was above the isoelectric point of CBD-*Ssp* DnaB-hEGF (The predicted pI = 5.70), therefore it was sufficient to disturb the stability of IBs.

### 3.3 Refolding and self-cleavage of solubilised CBD-*Ssp* DnaB-hEGF

**Figure 4:**
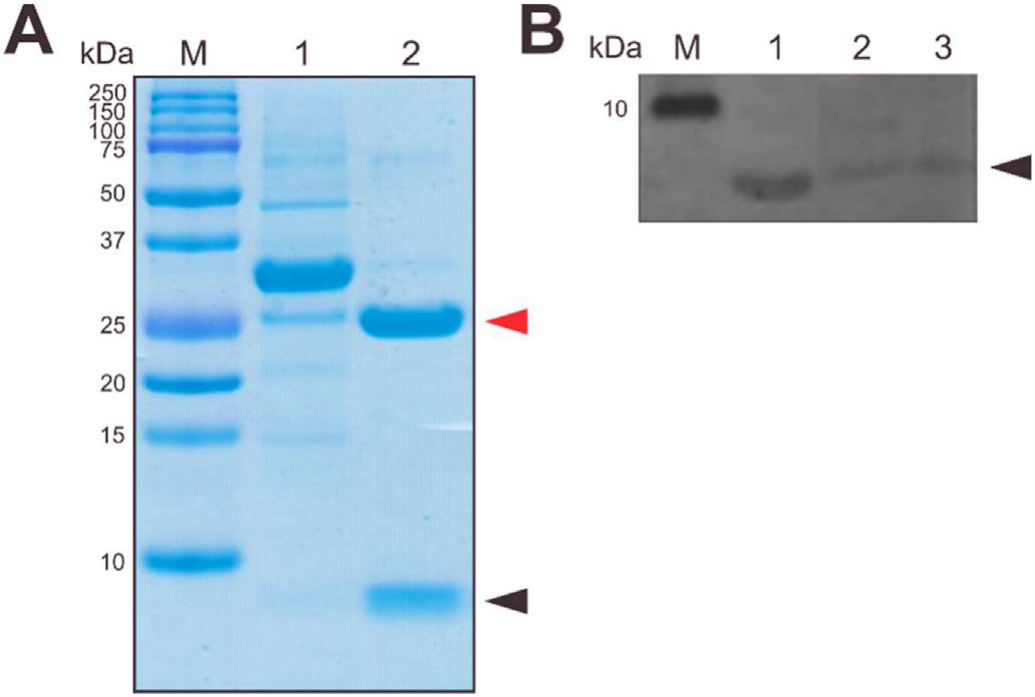
Oxidative refolding of solubilised CBD-*Ssp* DnaB-hEGF followed by intein cleavage. The glutathione-based redox system was added to refolding buffer and intein cleavage was induced by lowering the pH. (A) Tricine SDS-PAGE analysis of refolded hEGF. Lane M, protein marker (MW indicated on the left side); Lane 1, solubilised CBD-*Ssp* DnaB-hEGF; Lane 2, refolded hEGF. (B) Western blot analysis of refolded protein mixtures. The mouse anti-hEGF monoclonal antibody as primary and AP-conjugated anti-mouse IgG antibody as secondary was used to detect the presence of hEGF. Lane 1, commercial hEGF (1 mg/mL); Lane 2, mixture of refolded protein; Lane 3, heat-treated mixture of refolded protein. The black arrow corresponds to hEGF, while red arrow corresponds to CBD-*Ssp* DnaB. The original, non-cropped gels were provided in supplementary data as Figure S3 & S4.

Solubilised CBD-*Ssp* DnaB-hEGF was refolded by dilution, followed by *Ssp* DnaB mini-intein cleavage by dialysis. A mixture of GSH and GSSG was added to refolding buffer to provide the redox system for oxidative refolding and pH shifting was conducted during dialysis to induce splicing of *Ssp* DnaB mini-intein. Aggregation was observed due to the prolonged dialysis. SDS-PAGE analysis showed that the refolded hEGF was successfully released from the fusion protein (Figure 4 A), as confirmed by western blot analysis of the refolded protein mixtures, suggesting that hEGF was recognised by its antibody (Figure 4 B). These results indicated that refolded hEGF was obtained.

### 3.4 Characteristics of refolded hEGF

**Figure 5:**
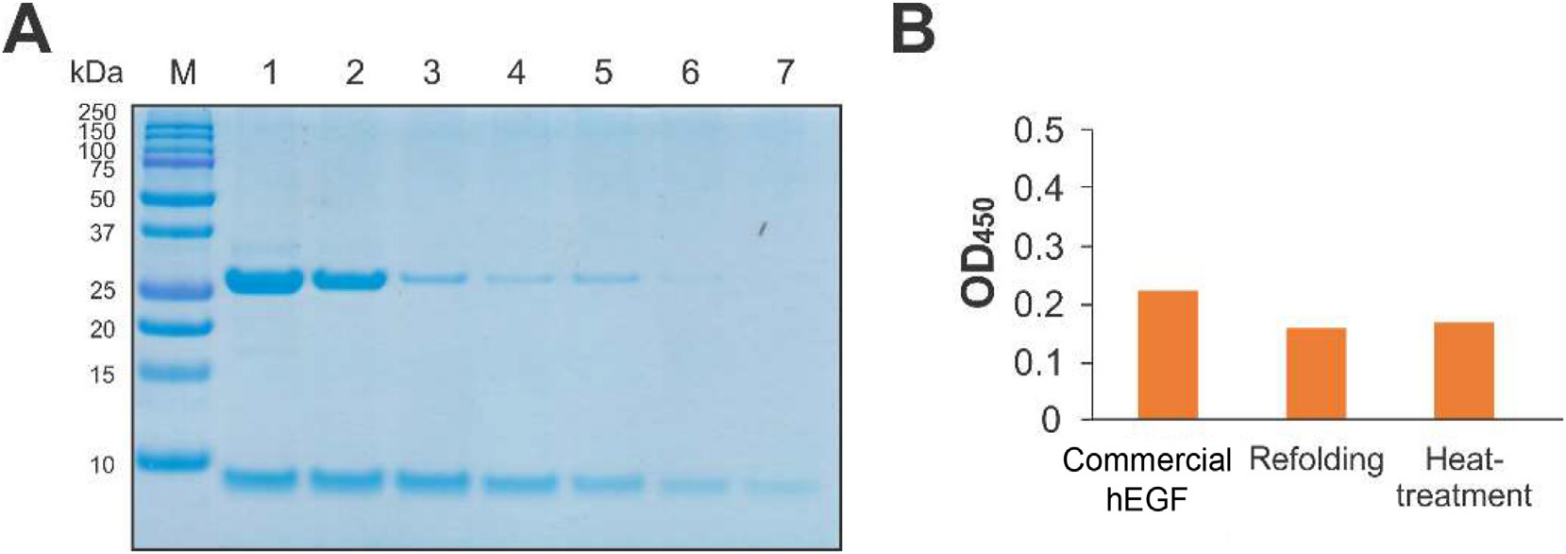
Analysis of refolded hEGF. Heat treatment and ELISA confirmed the correct folding of refolded hEGF. (A) Tricine SDS-PAGE analysis of heat-treated refolded hEGF. Lane M, protein marker (MW indicated on the left side); Lane 1, mixture of refolded protein; Lane 2–7, mixture of refolded protein after incubation at 60 °C, 65 °C, 70 °C, 75 °C, 80 °C and 85 °C. (B) Binding capacity of refolded hEGF measured by sandwich ELISA. Absorbance indicated that the refolded hEGF bound to the immobilised anti-hEGF.

**Figure 6:**
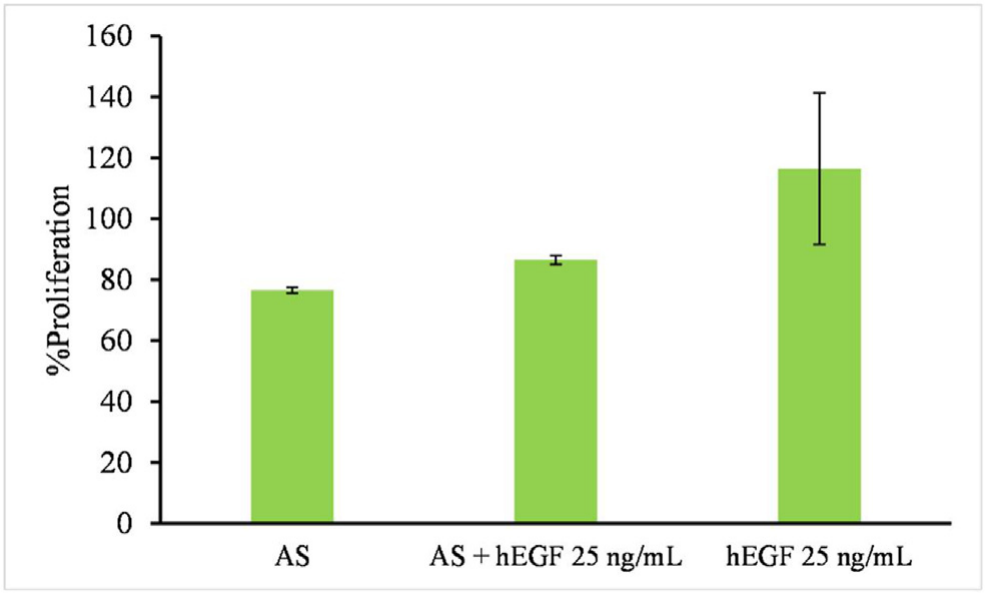
Keratinocyte proliferation percentage treated with autologous serum (AS), 25 ng/mL hEGF and the combination of both (AS + hEGF 25 ng/mL). The cell treated with hEGF alone exhibit higher percentage, indication proper protein folding was established. The data are means of triplicate readings; error bar was presented (Maksum et al., 2021).

hEGF is heat-resistant, even in boiling water (Cohen and Carpenter, 1975), therefore, the heat resistance properties of refolded hEGF were examined showing that the refolded hEGF possessed heat-resistant properties (Figure 5 A). Sandwich ELISA was performed to examine the binding capacity of hEGF, confirming that refolded hEGF bound to the immobilised anti-hEGF monoclonal antibody (Figure 5 B). The low amount of detected hEGF reflecting the inefficient refolding process, thus optimization of refolding process should be carried out in further study. The results indicated that hEGF was correctly folded, showing heat-resistant properties and binding capacity to a specific antibody. MTT assay was performed against keratinocyte cells to investigate the hEGF activity in stimulating cell proliferation. The hEGF performance was compared to treatment with autologous serum (AS) and the combination of both (Maksum et al., 2021). The autologous serum was naturally containing around 0.5 ng/mL EGF which the actual concentration was dependent to each persons (Geerling et al., 2004; Quinto et al., 2008). Keratinocyte treated with hEGF were shown to be able to stimulate cell proliferation (Figure 6). The proliferation percentage is even higher compared to other treatments.

### 3.5 Wound healing activity

**Figure 7:**
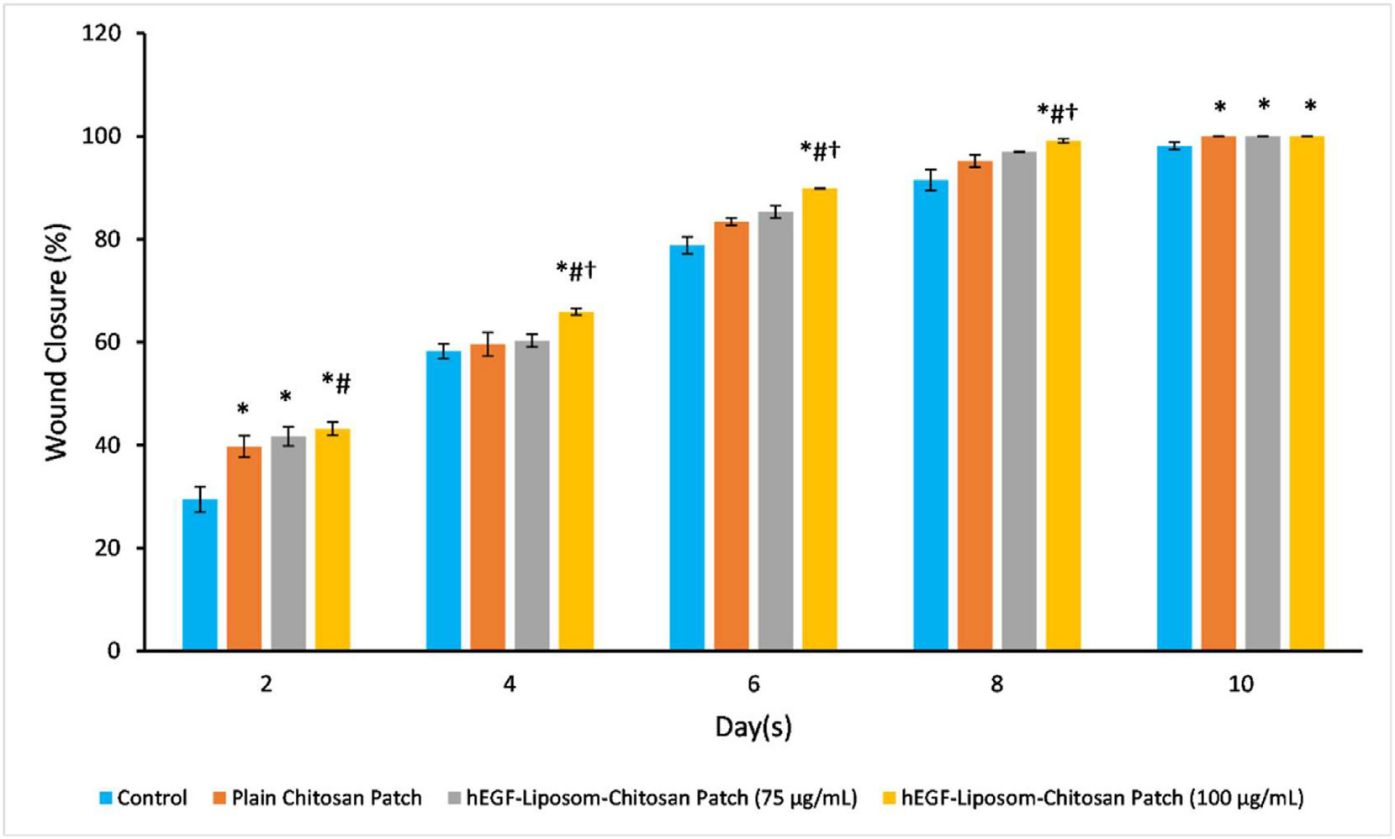
Refolded EGF activity in inducing wound closure. The refolded EGF was encapsulated in liposomal vesicles and formulated as thin-film chitosan. The percentage of day-by-day wound closure for each treatment group is presented. The symbols represent the significance of each groups, (∗) significantly higher than control (*p* < 0.05); (#) significantly higher than the plain chitosan patch (*p* < 0.05); (†) = significantly higher than the hEGF-liposome-chitosan patch (75 μg/mL) (*p* < 0.05). Data are the mean of quadruple readings with error bars.

**Figure 8:**
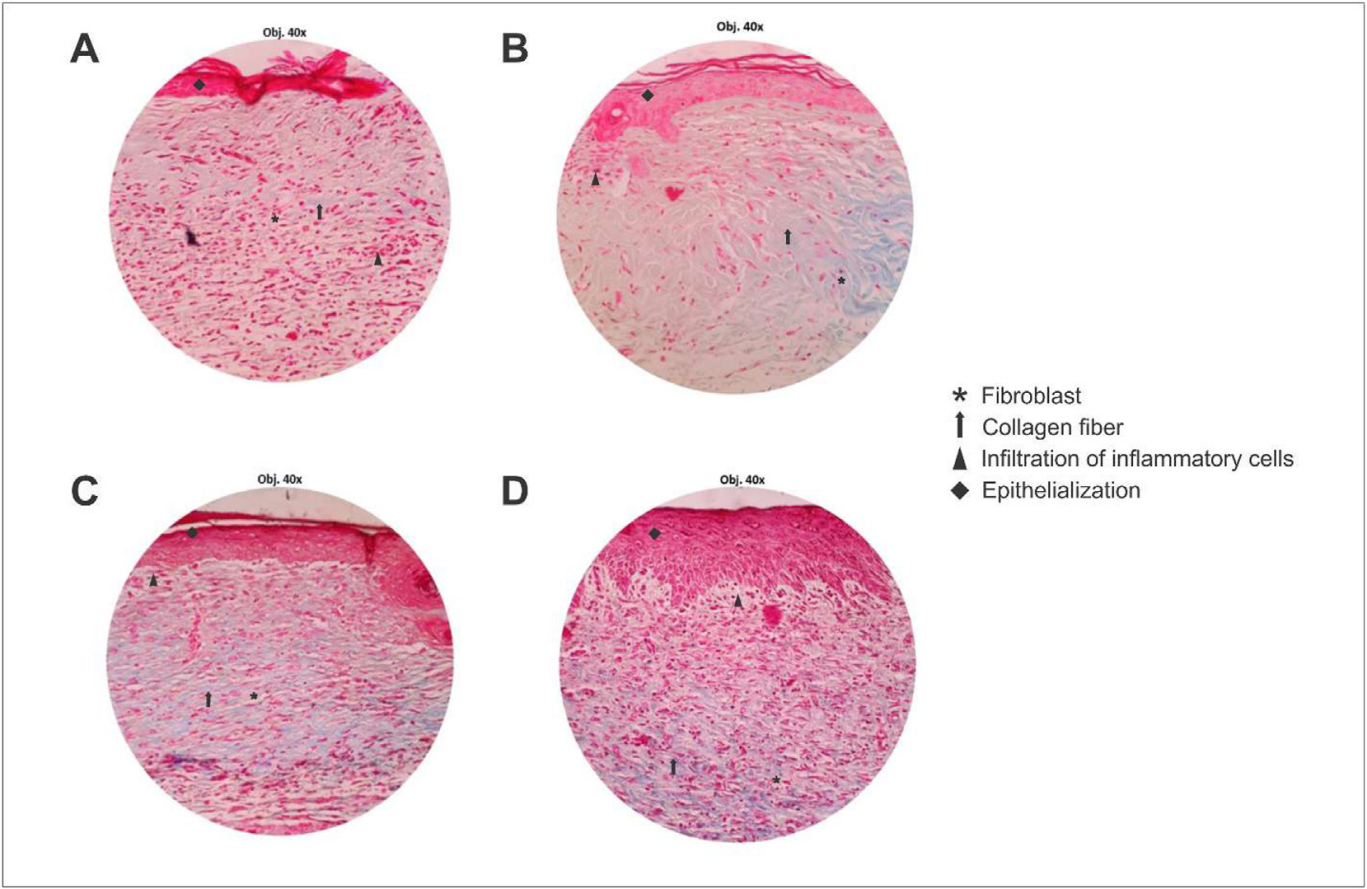
Histopathological analysis of mice skin stained with Mallory-azan at 400x magnification: (A) control group, (B) plain chitosan patch group, (C) hEGF-liposome-chitosan patch (75 μg/mL) and (D) hEGF-liposome-chitosan patch (100 μg/mL).

The refolded EGF was encapsulated into liposomes and incorporated with chitosan to form a thin-film formulation. The progression of the wound healing process was reflected as increasing wound closure (Figure 7). The mice treated with hEGF-liposome-chitosan (100 μg/mL) demonstrated significantly higher wound closure percentage day-by-day, hence the addition of exogenous hEGF accelerated wound healing in mice. On the 8^th^ day, all treatments provided a higher wound closure percentage than the control group while in 10^th^ day all treatment have complete wound closure and significantly different from the control group. Mallory-azan staining to distinguish extracellular tissue components (Sriwidodo et al., 2020) revealed that the mice treated with the hEGF-liposome-chitosan patch had thick epithelial formation, equally fibroblasts, and better collagenisation (Figure 8), indicating that the refolded EGF induced wound closure.

## 4 Discussion

Intracellular expression of small proteins is susceptible to proteolysis which leads to low protein expression (Zhang et al., 2015), therefore, hEGF was expressed as a fusion protein to prevent hEGF proteolysis by cytoplasmic proteases. *Ssp* DnaB-hEGF was chosen as the fusion partner because it has splicing activity induced by lowering the pH (Chen et al., 2002; Ding et al., 2003). It is also advantageous because it does not require a specific protease which is generally expensive (Sun et al., 2005). The CBD-*Ssp* DnaB-hEGF fusion protein was found to be abundant as IBs when overexpressed in *E. coli* BL21(DE3). Reduced cytoplasmic conditions and chaperone limitation of *E. coli* do not facilitate proper hEGF folding leading to the formation of IBs (Gomes et al., 2016). In this case, the formation of IBs was advantageous because it prevents *in vivo* splicing of *Ssp* DnaB mini-intein which causes loss of hEGF (Mathys et al., 1999). IBs were isolated from lysed *E. coli* cells by sonication followed by centrifugation as IBs are easily separated from the other cellular component due to differences in density (Singh and Panda, 2005). Isolated IBs were contaminated with impurities like cell debris and cellular protein (Vallejo and Rinas, 2004), which may interfere with the following steps so washing was conducted to remove the contaminants (Singh et al., 2015). Purified IBs were obtained after several washing steps, with the addition of 1 M urea and Triton X-100 to help purify the IBs.

Since the existence of the native-like secondary structure was revealed, many researchers have developed mild solubilisation methods (Chura-Chambi et al., 2019; Datta et al., 2013; Patra et al., 2000; Singh et al., 2008, 2012). It is assumed that by retaining the native-like secondary structure, it will be easier to fold the protein into its native form, leading to increased protein recovery from the IBs (Yon et al., 2018). In this study, we utilised a single freeze-thawing process as described by Qi et al. (2015) with some modifications. The IBs were solubilised by freeze-thawing in the presence of urea, as urea helps to disturb the hydrophobic interaction that stabilises the IBs (Patra et al., 2000). In the freeze-thawing method, the low concentration of urea was sufficient to disturb the hydrophobic interactions, while a high concentration of urea was needed for solubilisation by the urea denaturing method. The freezing process causes protein stress due to the formation of ice crystals (Cao et al., 2003), helping to separate proteins so that an even lower urea concentration can be used to solubilise IBs (Qi et al., 2015). The solubility of CBD-*Ssp* DnaB-hEGF in Tris buffer in the presence of 2 M urea by the freeze-thawing method was comparable to the urea-denaturation method in Tris buffer in the presence of 8 M urea. The effect of buffer pH on IBs solubilisation depends on the protein characteristics (Singh et al., 2008), therefore, the effect of Tris buffer pH on the solubilisation of CBD-*Ssp* DnaB-hEGF was also examined. For the urea denaturing method, the increased pH helped solubilise more CBD-*Ssp* DnaB-hEGF, while it did not affect IBs solubilisation in the freeze-thawing method. The alkaline pH which is far from the isoelectric point plays a role in destabilising IBs in the presence of a low concentration of urea (Patra et al., 2000; Singh et al., 2008).

The refolding steps are crucial for restoring the solubilised protein structure to the native form (Singh et al., 2015; Yamaguchi et al., 2013). As urea was removed by a dilution-based method, the solubilised protein started to refold. This method was chosen because the procedure is simple and solubilised protein was diluted with denaturant-free refolding buffer (Yamaguchi et al., 2013). We performed oxidative refolding to form correct disulphide bond of hEGF. Incorrect disulphide bond was reduced by addition small concentration of *β*-mercaptoethanol during solubilization step (Vallejo and Rinas, 2004). The widely used oxido-shuffling agent, glutathione in reduced and oxidised form was chosen to help the formation of correct disulphide bonds (Gieseler et al., 2018; Thomson et al., 2012), as it speeds up the bond formation of hEGF (Chang et al., 2001). *Ssp* DnaB mini-intein cleavage was performed by lowering the pH during dialysis (Yosua et al., 2021) to induce cyclisation of Asp154 at the C-terminal of *Ssp* DnaB mini-intein through a relay system involving some amino acid residues (Ding et al., 2003). It causes mini-intein cleavage and release of hEGF. The hEGF was correctly folded as evidenced by heat-resistant properties and binding capacity on ELISA. The refolded hEGF heat-resistant properties are maintained by the β-sheet structure stabilised by three native disulphide bonds (Yan et al., 2010). The refolded hEGF also bound to a specific antibody demonstrating correct folding on the epitope region of hEGF (Park et al., 2018). Furthermore, *in vitro* keratinocyte proliferation along with *in vivo* wound healing and histopathology confirmed the bioactivity of the refolded EGF.

## 5 Conclusion

This report describes a protocol for the recovery of refolded hEGF from bacterial IBs by mild solubilisation and oxidative refolding. Freeze-thawing was used to solubilise CBD-*Ssp* DnaB-hEGF in Tris buffer containing 2 M urea and hEGF was successfully released through oxidative refolding using a glutathione-based redox system followed by intein cleavage. Furthermore, the refolded hEGF was correctly folded and exhibit its bioactivity, indicating that the method is suitable for the high recovery of refolded hEGF from bacterial IBs.

## Declarations

### Author contribution statement

Iman Permana Maksum: Conceived and designed the experiments; Analyzed and interpreted the data; wrote the paper.

Yosua Yosua: Conceived and designed the experiments; Performed the experiments; Analyzed and interpreted the data; Wrote the paper.

Ahmad Nabiel: Analyzed and interpreted the data; Wrote the paper.

Riyona Desvy Pratiwi: Performed the experiments; Analyzed and interpreted the data.

Sriwidodo Sriwidodo: Conceived and designed the experiments; Analyzed and interpreted the data; Contributed reagents, materials, analysis tools or data; Wrote the paper.

Ukun M.S. Soedjanaatmadja: Contributed reagents, materials, analysis tools or data; Wrote the paper.

### Funding statement

This work was supported by Penelitian Dasar (PD) Grant (No 1207/UN6.3.1/PT.00/2021) and Academic Leadership Grant (No 1427/UN6.3.2/LT/2020) from the Directorate General of Research and Development Strengthening, Ministry of Technology Research and Higher Education, Republic of Indonesia.

### Data availability statement

Data included in article/supplementary material/referenced in article.

### Declaration of interests statement

The authors declare no conflict of interest.

### Additional information

No additional information is available for this paper.

## Appendix A Supplementary data

Supplementary Material.docx Supplementary Material.docx
