# Supplementary material for: Refolding of bioactive human epidermal growth factor from E. coli BL21(DE3) inclusion bodies & evaluations on its in vitro & in vivo bioactivity
Source: Heliyon. 2022 Apr 18;8(4):e09306. doi: 10.1016/j.heliyon.2022.e09306 (PMC9039848; doi:10.1016/j.heliyon.2022.e09306)
Supplement: Supplementary Material.docx [file mmc1.docx]

**Supplementary Data**

**
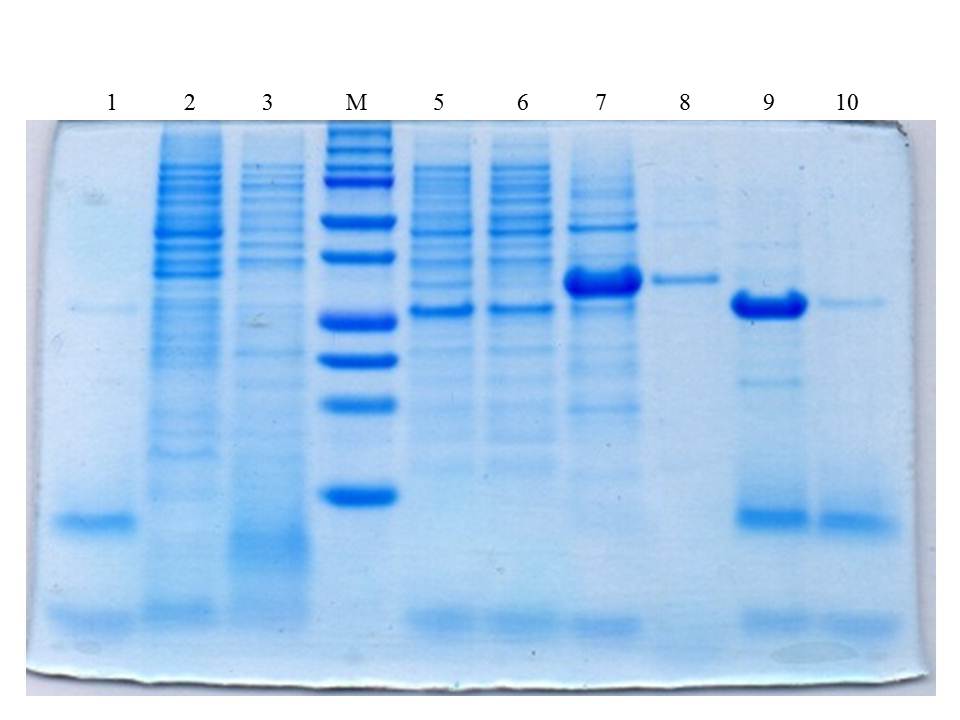
**

**Figure S1**. Tricine SDS-PAGE analysis of CBD-*Ssp* DnaB-hEGF expressionas IBs in *E. coli* BL21(DE3) related to Figure 1A. Lane M, protein marker; Lane 1, heat treatment cell lysate; Lane 2, protein aggregate; Lane 3, protein aggregate; Lane 5, induced cell lysate; Lane 6, cytoplasmic fraction of induced cells; Lane 7, un-purified IBs of induced cells; Lane 8, aggregate cell lysate; Lane 9, aggregate cells lysate; Lane 10, heat treatment cells lysate.


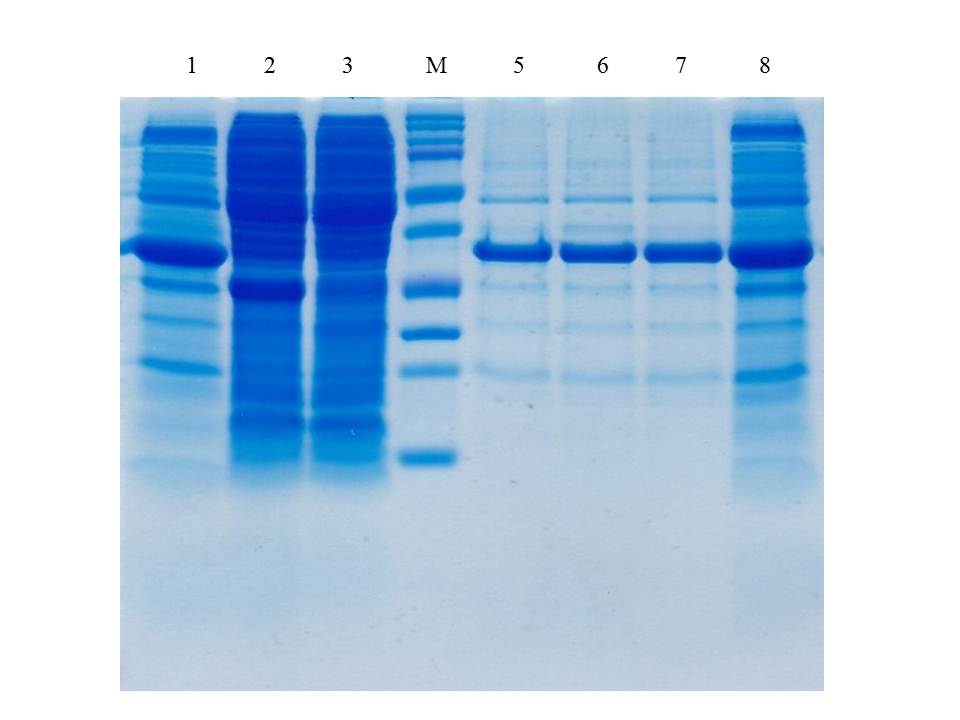


**Figure S2**. Tricine SDS-PAGE analysis of CBD-*Ssp* DnaB-hEGF expressionas IBs in *E. coli* BL21(DE3) related to Figure 1B. Lane M, protein marker; Lane 1, un-purified IBs; Lane 2, induced cell lysate; Lane 3, non-induced cell lysate; Lane 5, purified IBs; Lane 6, purified IBs; Lane 7, purified IBs; Lane 8, un-purified IBs.

**
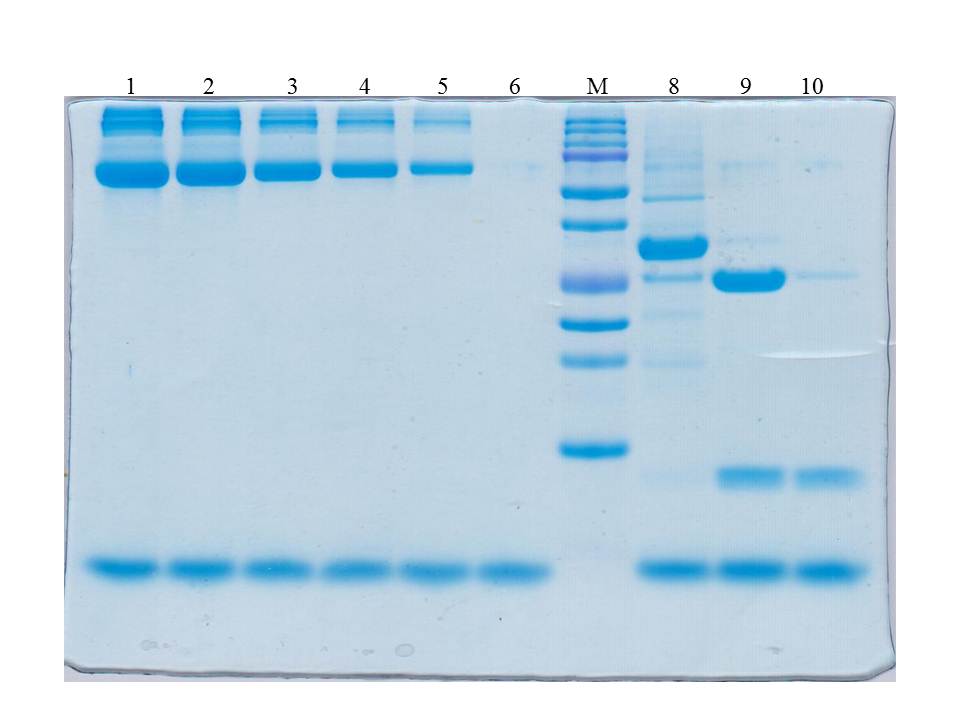
**

**Figure S3**. Tricine SDS-PAGE analysis of refolded hEGF related to Figure 4A. Lane M, protein marker; Lane 1-6, BSA (0.8, 0.6, 0.4, 0.2, 0.1 mg/ml); Lane 8, solubilised CBD-Ssp DnaB-hEGF; Lane 9, refolded hEGF; Lane 10, heat treatment cell lysate.

**
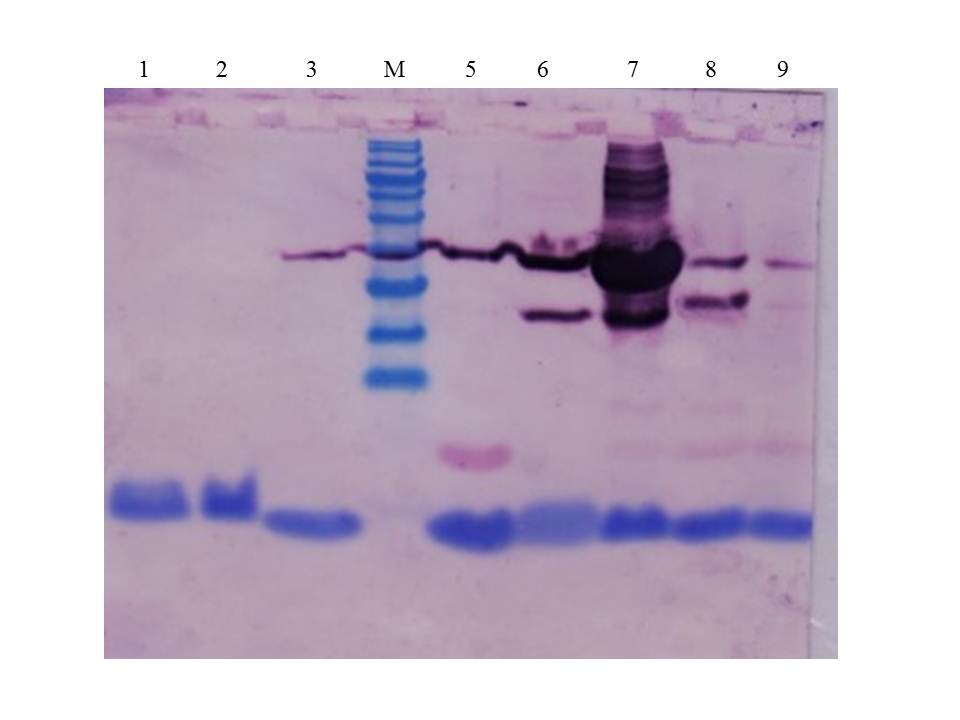
**

**Figure S4**. Western blot analysis of refolded protein mixtures related to Figure 4B. The mouse anti-hEGF monoclonal antibody as primary and AP-conjugated anti-mouse IgG antibody as secondary was used to detect the presence of hEGF. Lane M, protein marker; Lane 1, medium; Lane 2, periplasm; Lane 3, purified hEGF; Lane 5, commercial hEGF (1 mg/mL); Lane 6, lysate cell; Lane 7, solubilization IBs; Lane 8, mixture of refolded protein; Lane 9, heat-treated mixture of refolded protein.
